# Textbook of Melanoma

**DOI:** 10.1038/sj.bjc.6601865

**Published:** 2004-05-26

**Authors:** R Hubner, M Gore

**Affiliations:** 1 Department of Medicine, The Royal Marsden Hospital, Fulham Road, London SW3 6JJ, UK

This major new text is a well-presented and comprehensive review of melanoma. This first edition is presented in hardback with over 650 pages of text divided into 14 sections, which are further subdivided into 64 chapters. It has 94 contributors who practice in 14 different countries and therefore represents a truly global overview of current practice. Despite its size, the book is easy for the reader to navigate through due to its simple layout and well-structured chapters. It also benefits from over 300 colour illustrations, photographs, and line drawings, and many helpful tables and information boxes. The index is extensive making specific information easy to find and all chapters are well referenced.

The ‘Textbook of Melanoma’ includes sections on the basic biology of the pigment cell system, the aetiology, prevention, and screening of melanoma, and histopathology, classification, staging, and prognosis. It also covers diagnostic methods, the management of primary cutaneous melanoma, regional lymph nodes, and locally recurrent melanoma, as well as the diagnosis and systemic therapy of metastatic disease. There is also a useful chapter on primary melanoma in noncutaneous sites, and a review of current published guidelines.

All chapters include a historical perspective, detailed information regarding current understanding and best practice and where appropriate, challenges to the legitimacy of current practice with possible alternatives. For example, the chapter on staging systems for cutaneous melanoma describes early staging systems used from the 1950s, the application of the TMN staging system to melanoma in the 1960s, and the development of microstaging and use of Clark level and Breslow depth. The 1997 AJCC staging system for cutaneous melanoma is described in detail, and then critically analysed with proposed changes.

Owing to its comprehensive nature, this text will be of interest to oncologists, dermatologists, and surgeons with an interest in skin cancer. All three editors are surgeons, and this is reflected in the detailed description of surgical procedures and operative techniques, along with colour photographs and illustrations.

Any oncology text will be unable to present the latest developments in a rapidly advancing field; however, the chapter on new anticancer agents discusses new cytotoxic agents, cyclin-dependent kinase inhibitors, signal transduction inhibitors, and antiangiogenesis agents, and includes data from papers and abstracts up to and including year 2000.

The ‘Textbook of Melanoma’ was clearly an ambitious project, and the editors should be congratulated on producing this important comprehensive reference book.

